# A Polarization-Insensitive Broadband Terahertz Absorber Using Patterned Graphene

**DOI:** 10.3390/nano12213763

**Published:** 2022-10-26

**Authors:** Maixia Fu, Jinyi Wang, Shaoshuai Guo, Zhaoying Wang, Pengxu Yang, Yingying Niu

**Affiliations:** 1 Key Laboratory of Grain Information Processing and Control (Henan University of Technology), Ministry of Education, Zhengzhou 450001, China; 2 Henan Key Laboratory of Grain Photoelectric Detection and Control, Henan University of Technology, Zhengzhou 450001, China; 3 College of Information Science and Engineering, Henan University of Technology, Zhengzhou 450001, China

**Keywords:** absorber, terahertz, graphene, polarization insensitive, broadband

## Abstract

A polarization-insensitive broadband terahertz absorber with a sandwich structure of metal–dielectric-graphene is designed and simulated. The graphene is patterned as an array of graphene square blocks with circular apertures. The results of the simulations and theoretical analysis show that the absorption exceeds 99% from 0.93 to 1.65 THz while 90% from 0.80 to 1.87 THz, and a broad relative bandwidth of 80.2% is achieved. The absorption performance is passively enhanced by altering physical dimensions of the graphene pattern and actively adjusted by changing the chemical potential of graphene. When the chemical potential increases from 0.1 eV to 0.7 eV, the corresponding terahertz absorption increases from 59.1% to 99%. The mechanism of absorption is disclosed by analyzing the impedance matching theory and distribution of electric-field intensity. In addition, different polarization modes and incident angles are also studied. The proposed absorber has the superiorities of broad relative bandwidth, high absorption rate, polarization insensitivity, and a wide incident angle, which offers some potential applications in the field of terahertz technology such as imaging, detection, and cloaking.

## 1. Introduction

The terahertz frequency band located in the transition region from electronics to photonics in an electromagnetic wave is generally regarded as 0.1–10 THz. Terahertz has great application prospects in biological diagnosis, detection sensing, security inspection, wireless communication, and other fields due to its unique frequency band characteristics [1]. It is known that an integrated terahertz system requires many components with different functions. An absorber is one kind of the important components and has been widely used in many applications, such as cloaking, probing, and imaging [2]. In recent years, terahertz absorbers have been studied by more and more scholars. Due to the lack of natural absorbing materials in the terahertz gap, metamaterials are regarded as an effective solution. The micro- or nanostructures of metamaterials can control the incident light scattering in a specific way and can produce strong coupling effects with the electromagnetic wave at the resonant frequency to improve the performance of the terahertz absorber. Most of the early absorbers were made of metal metamaterials. However, in the terahertz frequency range, metal is considered an ideal conductor and can reflect almost all terahertz waves. Resonance absorption formed by metal metamaterials often has a very narrow bandwidth. On the other hand, once the structural parameters of this type of absorber are given, the resonant frequency and absorption rate are difficult to change again, which is not conducive to practical application [3,4]. Therefore, it is crucial to design an absorber that has the properties of broad operation bandwidth and is dynamically tunable.

Graphene, a single layer of carbon atoms, was the first two-dimensional material discovered [5] and has been regarded as a band-gap semiconductor. Previous studies have shown that by external voltage, optical pump, or chemical doping, the carrier concentration in graphene can be effectively changed, thus leading to the change of graphene conductivity [6,7,8]. Recently, graphene has become a hot spot in the studies of tunable terahertz absorbers. In 2017, Dong et al. suggested a broadband absorber that consisted of stacking multilayer graphene. The absorption achieved 90% in the terahertz band of 2.58–3.56 THz with a relative bandwidth of 31.9%. The proposed structure exhibited effective absorption performance when the incident angle changed from 0° to 50°. The device was ultrathin with a thickness of less than λ_0_/21 [9]. Using a stacking method, the absorption band was widened, but the structure was a little complex. In 2018, Huang et al. suggested an absorber consisting of a classical sandwich structure: a target-patterned graphene resonator, TOPAS polymer (a cyclic olefin copolymer, which has very low loss and low material dispersion in the terahertz regime), and gold. An absorption of more than 90% was obtained from 0.95 to 2.52 THz with a relative bandwidth of 90.4%. Due to the symmetry of the structure, the absorber had the properties of polarization insensitivity. The absorption remained more than 75% over wide incidence angles up to 60° for TE mode and 75° for TM mode [10]. The proposed absorber had a single-layer graphene pattern, which can be easily replicated. In 2019, Liu et al. presented an ultra-broadband absorber that was arranged in a sandwich structure: an array of metal cylinders, two-layers of graphene, and a metal substrate at the bottom. The absorption was greater than 80%, and the bandwidth was about 7.10 THz. The broadband absorption was achieved with two different resonances in graphene, and a high absorption rate was achieved with metallic cylinders, which were used to fulfill impedance matching in the free space [11]. The absorber had ultra-broad absorption bandwidth, but the absorption rate was not relatively high. In 2020, Wu et al. suggested a terahertz absorber using multilayer graphene grating and Si dielectric material. The maximum absorption was dynamically tunable to reach 99% with a bandwidth of 0.15 THz from 1.015 to 1.165 THz [12]. Although the absorber had a high absorption efficiency, the absorption bandwidth was relatively narrow. In 2021, Nickpay et al. studied an absorber using patterned graphene, which was insensitive to polarization. The graphene pattern was on the top layer consisting of two concentric rectangular rings and a crossbar. Within 2.47–2.90 THz and 2.39–2.93 THz, the bandwidths with coefficients of 0.9 and 0.7 were 16% and 20.3%, respectively [13]. The proposed device had the properties of independent polarization, but the structure was relatively complex and difficult to fabricate. In 2022, Yu et al. demonstrated a polarization-insensitive broadband terahertz absorber using graphene metamaterial constructed with three concentric circinate strips and a relatively complex octagon arrangement; the maximum absorption was over 90% from 1.027 to 1.958 THz with a relative bandwidth of 62.8% [14]. However, the structure of the absorber is complicated. From the above findings, it is clear that to realize high absorption rates and broad absorption bandwidth, most graphene-based absorbers use multilayer structures and complex patterns, which are not easy to fabricate and may affect the compactness of the devices. Meanwhile, polarization insensitivity is often achieved with the help of structural symmetry. Therefore, studies on absorbers that simultaneously have the properties of simple structures for easy fabrication, high absorption rates, a broad relative bandwidth, a wide-angle absorption, and dynamic tunability are still needed for further investigation of the practical applications.

In this study, a polarization-insensitive broadband tunable terahertz absorber is designed and simulated. The absorption acquired exceeds 99% from 0.93 to 1.65 THz and 90% from 0.80 to 1.87 THz. By optimizing the length and width of graphene blocks and the radius of the graphene circular aperture, the absorption properties could be enhanced. By adjusting the thickness of the middle dielectric layer, the absorption rate and bandwidth could be tuned. By varying the chemical potential of graphene from 0.1 eV to 0.7 eV, the maximum absorption could be dynamically tuned can increased from 59.1% to 99.6%. The mechanism of this high broadband absorption phenomenon is disclosed by analyzing the electric-field distribution. The excitation of localized surface plasmon resonance (LSPR) in graphene is responsible for the absorption performance. In addition, the polarization-insensitivity phenomenon occurs because of the geometric symmetry of the designed structure. Thus, the proposed graphene-based terahertz absorber can be applied to terahertz cloaking, switching, modulators, etc.

## 2. Design and Methods

**Figure 1:**
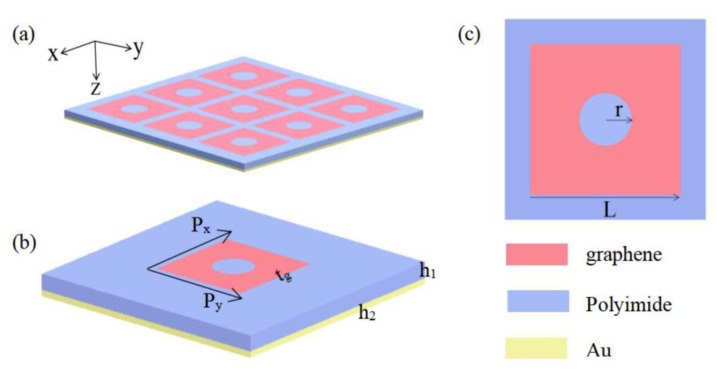
Structure of the proposed absorber. (**a**) Schematic view; (**b**) unit cell; (**c**) top view (x-y plane).

The schematic view of the designed absorber is displayed in Figure 1, which is a sandwich structure: an array of graphene square blocks with circular apertures, a polyimide layer as a spacer in the middle, and a gold plate as a substrate to block terahertz transmission.

The structural parameters of the proposed absorber are listed in Table 1. The conductivity of the bottom gold plate is $\sigma =4.56\times 10^{7}\;S/m$. The loss of polyimide is very low at the terahertz frequency, and its relative dielectric constant is about 2.4.

In the calculations, graphene was considered a very thin dielectric film with a thickness of $t_{g}=1\;nm$. The relative dielectric constant of graphene is described as $\varepsilon_{g}=1+\frac{i\sigma_{g}}{\omega \varepsilon_{0}t_{g}}$, where $\sigma_{g}$ means the surface conductivity of graphene and can be deduced from the Kubo formula [15]:

(1) $$\begin{array}{l} \sigma (\omega ,\mu_{c},\tau ,T)=\sigma_{\mathrm{int}er}+\sigma_{\mathrm{int}ra}\\ \;\;\;\;\;=\frac{je^{2}(\omega -j\tau^{-1})}{\pi \hbar^{2}}\times [\frac{1}{{\left(\omega -j\tau^{-1}\right)}^{2}}\int_{0}^{\infty }\frac{\partial f_{d}(\varepsilon )}{\partial \varepsilon }-\frac{\partial f_{d}(-\varepsilon )}{\partial \varepsilon }d\varepsilon -\int_{0}^{\infty }\frac{f_{d}(-\varepsilon )-f_{d}(\varepsilon )}{{\left(\omega -j\tau^{-1}\right)}^{2}-4{\left(\frac{\varepsilon }{\hbar }\right)}^{2}}d\varepsilon ] \end{array}$$

where e is the electronic charge, ℏ is the approximate Planck constant, ω is the angular frequency, $f_{d}\left(\varepsilon \right)$ is the Fermi–Dirac distribution, and τ is the momentum relaxation time.

According to Formula (1), there are two factors for the surface conductivity of graphene. The former originates from interband transition and is represented by the symbol $\sigma_{inter}$. The latter is caused by intraband transition and is marked with symbol $\sigma_{intra}$. Considering the room temperature and terahertz frequencies here, the Fermi energy in graphene is usually far more than half of the photon energy. Thus, the conductivity of graphene is largely affected by intraband transition, and the influence of interband transition on it almost small enough to be ignored. The process of intraband electron–photon scattering gives a Drude-like dispersion. Therefore, the calculation of conductivity in graphene can be simplified by the following formula [16]:

(2) $$\sigma_{g}\approx \sigma_{\mathrm{intra}}(\omega )=\frac{e^{2}\mu_{c}}{\pi \hbar^{2}}\frac{i}{\omega +i\tau^{-1}}$$

where $\mu_{c}$ is the chemical potential of graphene, and the initial value of momentum relaxation time is defined as τ=1 ps.

**Figure 2:**
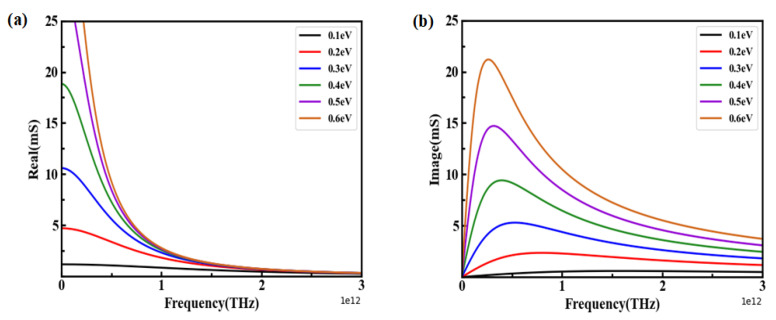
Conductivity with different $\mu_{c}$. (**a**) Real part; (**b**) imaginary part.

According to Formula (2), Figure 2a,b depict the real part (Re) and imaginary part (Im) of the $\sigma_{g}$ increase with $\mu_{c}$ increasing at the low terahertz frequencies. Once the $\mu_{c}$ was fixed, the real part (Re) of $\sigma_{g}$ decreased sharply until it approached nearly zero and remained stable at high terahertz frequencies. However, as the chemical potential $\mu_{c}$ increased, the imaginary part (Im) first rose and then fell, leading to a peak. This was due to the resonance absorption of the electron transition. In other words, the real part (Re) and imaginary part (Im) of $\sigma_{g}$ determined the amplitude modulation and spectral shift of the absorption spectra [17]. Based on these theoretical analyses, it is possible to realize the dynamic modulation of absorption spectra via adjusting the chemical potential of graphene.

**Figure 3:**
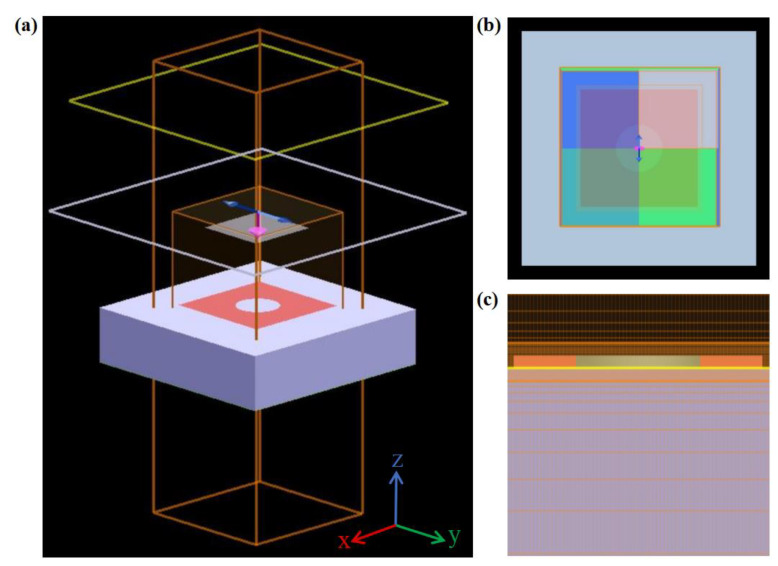
FDTD simulation of the designed absorber. (**a**) Perspective view of the absorber; (**b**) view of the x-y plane; (**c**) part view of the y-z plane (the selected part here is to display the mesh grid clearly).

The properties of the designed absorber are analyzed with a commercial simulation software named FDTD solutions (The software is produced by Canadian Lumechanical Solutions, Vancouver, BC, Canada). Figure 3a shows the three-dimensional model established in the FDTD solutions. In the simulations, the plane wave was used as a light source, which normally occurs to the top of arrays. The electrical vector was parallel to the y-direction. The boundary condition of the z-direction was set to be a perfectly matched layer (PML). Figure 3b illustrates the view of the x-y plane. A symmetrical boundary condition was adopted for the x-direction, and antisymmetrical boundary conditions were adopted for the y-direction. Such settings could shorten the simulation time. Figure 3c shows the partial view of the y-z plane, where the override 2 nm mesh in the x- and y-direction and the 0.1 nm mesh in the z-direction were set to improve the accuracy of the pattern region, ensuring the simulation results could converge well.

The absorption can be expressed as A(ω)=1−T(ω)−R(ω), in which T(ω) represents the terahertz transmission, and R(ω) represents the terahertz reflection. In addition, both T(ω) and R(ω) can be expressed in terms of S-parameters as T(ω)=|*S*_21_|^2^ and R(ω)=|S_11_|^2^, respectively, where $S_{21}$ and $S_{11}$ are the complex transmission coefficient and reflection coefficient, respectively. Because the substrate is metal, which can prevent the downward transmission of a terahertz wave, T(ω)=0 can be acquired. The absorption can then be simplified as A(ω)=1−R(ω)=1−|*S*_11_|^2^. Considering the impedance matching principle, when the equivalent input impedance matches the free space impedance, the absorption achieves the maximum yield, and the normalized equivalent input impedance can be described as follows [18]:

(3) $$Z(\omega )=\sqrt{\frac{{\left(1+S_{11}\right)}^{2}-S_{21}^{2}}{{\left(1-S_{11}\right)}^{2}-S_{21}^{2}}}$$

According to Formula (3), when $S_{11}=0$ and $S_{21}=0$, Z(ω) is equal to 1, and when the absorber matches the free space impedance, the maximum absorption can be obtained, and a nearly perfect absorption can be achieved.

## 3. Results and Discussion

### 3.1. Absorption Properties of the Proposed Absorber

**Figure 4:**
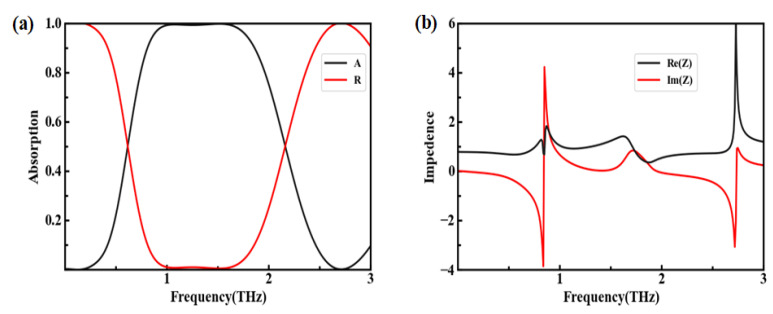
Absorption performance and equivalent impedance. (**a**) Absorption and reflection spectra; (**b**) real and imaginary parts of impedance Z.

Figure 4a shows the absorption and reflection spectra of the absorber. The related parameters are defined as p=33 μm (*P_x_* = *P_y_* = p), L=25 μm, $h_{1}=36\;\mu m$, $h_{2}=1\;\mathrm{nm}$, and $\mu_{c}=0.7\;\mathrm{eV}$. According to Formula (3), the absorption is only related to $S_{11}$ when $S_{21}$ is zero. Thus, the surface reflection needs to be minimized to improve the absorption performance. In our study, a high absorption of more than 99% was observed from 0.93 to 1.65 THz, and the bandwidth was 0.72 THz. Figure 4b shows real part Re(Z) and imaginary parts Im(Z) of the equivalent impedance of the device. The results revealed Re(Z) was very close to 1, and Im(Z) nearly approached zero in the scope of 0.80–1.87 THz, indicating that the broadband impedance matched. Therefore, the excellent absorption performance obtained was rooted in the impedance matching between the absorber and the free space, which minimized the terahertz reflection and maximized the terahertz absorption.

**Figure 5:**
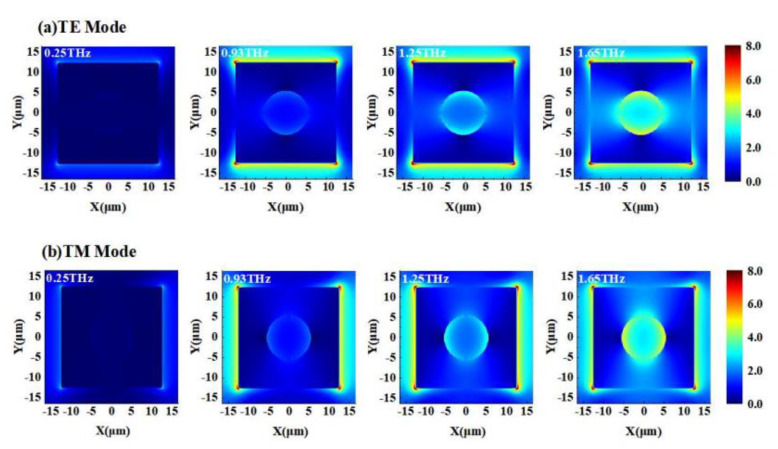
Distribution of electric-field. (**a**) TE mode; (**b**) TM mode.

Figure 5 shows the electric-field intensity in the x–y plane at 0.25 THz, 0.93 THz, 1.29 THz, and 1.65 THz in TE and TM polarization modes. When at 0.25 THz, the surface electric-field was very weak and had a uniform distribution inside the graphene square block. When at 0.93 THz, the aggregation effect of the electric-field appeared, and a strong electric-field was mainly concentrated in the upper and lower edges of the graphene square block for TE mode, while in the left and right edges for TM mode. The strongest electric field appeared in the four corners of the graphene square block, the second strongest electric field inside the circular aperture of graphene, and the weakest electric field inside the graphene block. The working mechanism of the absorber can be attributed to a typical absorption, which is realized by electrical and magnetic resonance. The electrical resonance mainly originated from the upper graphene pattern, while the magnetic resonance occurred between the graphene layer and the bottom metal layer. However, as the frequency increased (for example, at 1.29 THz) the electric-field began to extend from both edges of the graphene square block to the circular aperture, and a nearly 100% absorption was obtained. When at 1.65 THz, the electric field distribution inside and around the graphene circular aperture was further enhanced, leading to a perfect absorption. This can be explained by local surface plasmon resonance [19]. When the patterned graphene interacted with the terahertz wave, localized plasmons were resonantly excited in both edges of the graphene block and around the circular aperture structure. These two resonances produced a combined effect in the overlapping region and gave a probability of excellent absorption performance [20].

### 3.2. Effects of Structural Parameters on Absorption Properties

**Figure 6:**
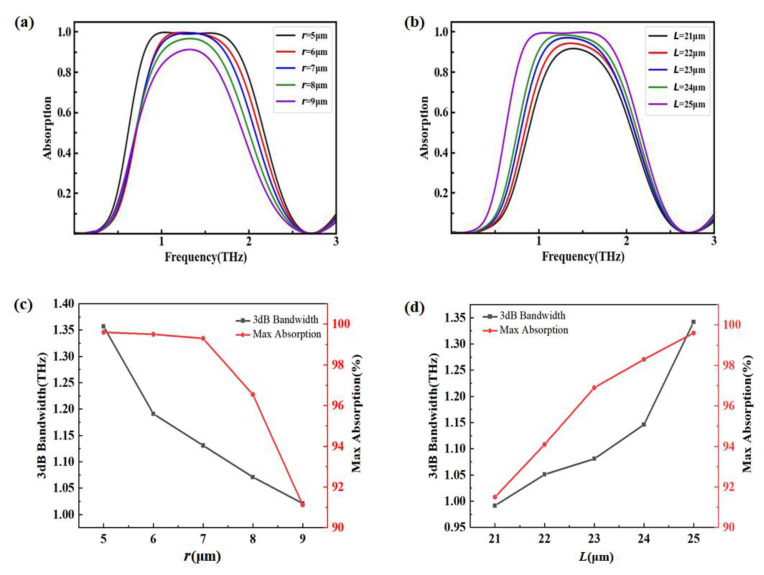
Absorption spectra with different geometrical parameters. (**a**) Different r; (**b**) different L; (**c**,**d**) 3 dB bandwidth and the maximum absorption with different r and L, respectively.

The influence of different geometrical parameters on absorption properties are illustrated in Figure 6. The related parameters were set as p=33 μm, L=25 μm, $h_{1}=36\;\mu m$, and $\mu_{c}=0.7\;\mathrm{eV}$ in the simulations. Figure 6a shows the absorption spectrum with different radii r. By increasing r from 5 μm to 9 μm, the absorption decreased and the bandwidth narrowed. When r=5 μm, the maximum absorption was 99.6%. When r=9 μm, the minimum absorption still reached 91.1%. The reason for these phenomena is that when the radius r increased, not only did the effective resonance length increase, but also the effective area of the graphene square block decreased. Therefore, the absorption showed a trend of gradually decreasing, and the bandwidth showed a trend of narrowing. To clarify further, the 3 dB bandwidth and the maximum absorption with different r are illustrated in Figure 6c. It is clear that both of the 3 dB bandwidth and the maximum absorption decreased with the increase of radius r.

The effect of the parameter L on the absorber’s performance was discussed, and the corresponding results are illustrated in Figure 6b. When L varied from 21 μm to 25 μm, the absorption increased from 91.7% to 99.6%, and the absorption bandwidth gradually became wider. As the length L increased, the effective area of the graphene square block enlarged, leading to a strong broadband absorption. In addition, the 3 dB bandwidth and the maximum absorption with different L were extracted and are plotted in Figure 6d. It shows that the 3 dB bandwidth and the maximum absorption increased with an increasing L. When L=21 μm, the maximum absorption was 91.5%, and the 3 dB bandwidth was 0.99 THz. When L=25 μm, the maximum absorption was 99.6%, and the 3 dB bandwidth was 1.34 THz.

**Figure 7:**
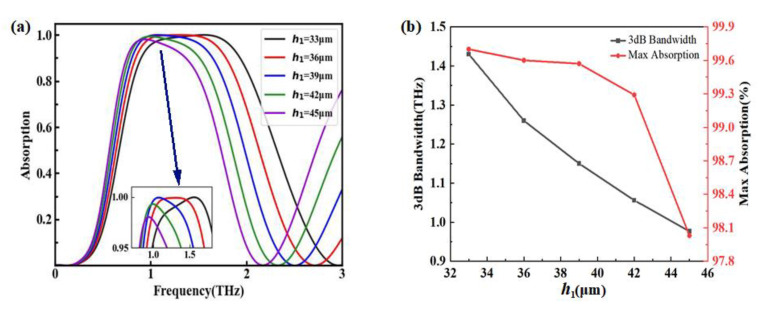
Absorption properties with different thickness of middle dielectric layer. (**a**) With different $h_{1}$; (**b**) 3 dB bandwidth and the maximum absorption with different $h_{1}$.

The influence of the middle dielectric layer with different thicknesses is depicted in Figure 7. In our simulations, polyimide with a refractive index of 1.53 was adopted as the middle dielectric layer, and $h_{1}$ represented the thickness. In Figure 7a, when $h_{1}$ changes from 33 μm to 45 μm, the absorption spectra tends to be red-shifted and broadband, while the maximum absorption shows very little change. In order to facilitate the analysis, 3 dB bandwidth and the maximum absorption with different $h_{1}$ are plotted Figure 7b. The maximum absorption decreased from 99.7% to 98.0% as the $h_{1}$ increased with a step of 3 μm, and the 3 dB bandwidth had an obvious trend of decreasing from 1.43 THz to 0.98 THz.

### 3.3. Effects of Chemical Potential of Graphene on Absorption Properties

**Figure 8:**
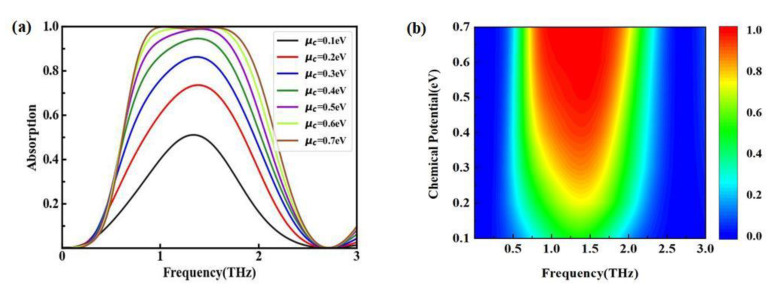
Absorption properties with different $\mu_{c}$. (**a**) Absorption spectra; (**b**) absorption contour view.

Whether the absorber has dynamic adjustability was one of the important criteria to judge its performance. As an excellent two-dimensional material, the conductivity of graphene has proven to be connected with its chemical potential $\mu_{c}$ and can be tuned by photodoping or voltage gating. Thus, by adjusting $\mu_{c}$, the operating frequency and performance of the device could be dynamically regulated. Figure 8a shows the absorption spectra with different $\mu_{c}$, and Figure 8b shows the absorption contour view. When $\mu_{c}\;$ increased from 0.1 eV to 0.7 eV, the maximum absorption increased from 59.1% to 99.6%, and the corresponding bandwidth widened. This was because the conductivity of graphene is related to the chemical potential, which is illustrated by Formula (2) above. When $\mu_{c}$ increased, the conductivity of graphene also showed a trend toward increasing, which originated from the increase in the density of the carriers. Once the chemical potential increased to a certain level, graphene behaved like a quasi-metal with high conductivity. In such a case, the incident terahertz wave could excite the localized surface plasmon resonance in graphene. The higher chemical potential may have caused a stronger resonance, thus leading to a broadband and high absorption.

### 3.4. Effects of Polarization and Incident Angle on Absorption Properties

**Figure 9:**
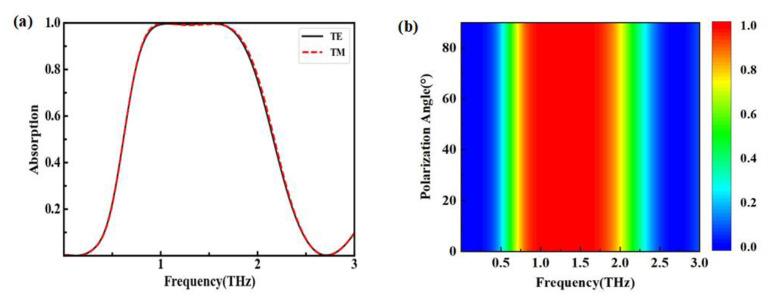
Absorption properties with different polarization. (**a**) Absorption spectra in TE and TM mode; (**b**) absorption contour view with different polarization angles in TE mode.

An important index parameter to evaluate the performance of an absorber is to see whether it can always retain good absorption performance under the conditions of different polarization modes, different polarization angles, and different incident angles [21,22]. The influence of different polarization modes and polarization angles are also illustrated in Figure 9. Figure 9a displays the absorption curves of TM and TE polarization modes, which are similar to each other. Figure 9b shows the absorption contour view with different polarization angles for TE mode. It is clear that the absorption was almost invariant when the polarization angle varied from 0° to 90°. Therefore, the absorber proposed here is polarization-insensitive. The reason is the high symmetry of the designed structure. The characteristic of polarization insensitivity offers the broadband absorber a robust function in practical applications.

**Figure 10:**
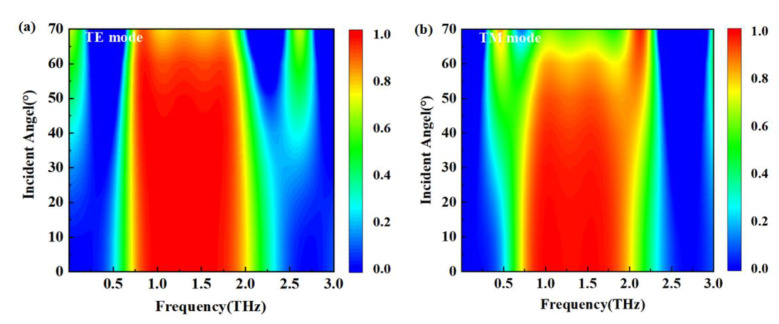
Absorption properties with different incident angles. (**a**) TE mode; (**b**) TM mode.

The influence of incident angles was also discussed. The related results are illustrated in Figure 10. It is clear that for a relatively wide range of incident angles, the absorber had an excellent absorption performance and broad bandwidth. For the TE mode in Figure 10a, the absorption remained quite high and maintained a fairly wide bandwidth when the incident angle was between 0° and 60°. As the incident angle exceeded 60°, terahertz absorption began to decrease. For the TM mode in Figure 10b, the absorption still maintained a high rate and a broad bandwidth, provided the incident angle did not exceed 40°. As the incident angle exceeded 40°, the absorption gradually decreased, and multiple peaks began to appear at about 60°, which might be due to the phase mismatch between absorption and reflection offset.

### 3.5. Comparison of Absorption Performance with Previous Absorbers

Finally, comparisons of our designed absorber to other previously reported absorbers were carried out, and the results are shown in Table 2 [23,24,25,26,27,28]. The absorber proposed here has four advantages. (1) The geometric structure is relatively simple, so the manufacturing process is not complicated. (2) A monolayer graphene is used, which is conducive to compactness. (3) The presented absorber has a relatively wide bandwidth (0.80–1.87 THz) of 80.15% while maintaining a high absorption (more than 90%). (4) The proposed absorber possesses properties of wide-angle absorption and insensitive polarization.

## 4. Conclusions

In conclusion, a simple structure of a graphene-based terahertz absorber is designed to achieve high absorption, broadband, and polarization insensitivity. The proposed device consists of a periodical array of graphene square blocks with circular apertures, a polyimide dielectric layer as a spacer, and a gold plate as a substrate. Terahertz absorption is more than 90% from 0.80 THz to 1.87 THz, and more than 99% from 0.93 THz to 1.65 THz. By optimizing the geometric size of the absorber and adjusting the chemical potential of graphene, the absorption could be modulated from 59.1% to 99.6%. The mechanism of the absorption properties is disclosed and analyzed. Moreover, the proposed absorber is polarization-insensitive and can achieve high broadband absorption in both TE and TM modes. It also performed well when the incident angle is in the range of 0–60°. Compared with the previously reported absorbers, this absorber has a simpler structure for easy fabrication and nearly perfect absorption, relative broadband, and active modulation, which can be used for various applications in terahertz cloaking, modulating, switching, sensing, etc.

## Figures and Tables

**Table 1:** Structural parameters of the proposed absorber.

Parameter Symbol	Physical Meaning
*P_x_*	Period of unit cell in x-direction
*P_y_*	Period of unit cell in y-direction
*L*	Length of graphene square block
r	Radius of circular aperture
$h_{1}$	Thickness of polyimide
$h_{2}$	Thickness of Au
$t_{g}$	Thickness of graphene

**Table 2:** Comparison with the published terahertz absorbers.

Reference	Bandwidth > 90%	Relative Bandwidth	Material	Max Incident Angle (°)
[23]	3.80–4.50 THz	16.8%	MoS_2_-SiO_2_-Metal	60
[24]	4–7 THz	54.55%	Al-Si_3_N_4_-VO_2_-Si_3_N_4_	40
[25]	1.87–4.19 THz, 0.73–8.70 THz	76.57%, 20.9%	VO_2_	-
[26]	4.8–5.56 THz	13.51%	Three-layer Graphene-Al_2_O_3_-Gold	60
[27]	6.98–9.10 THz	26.12%	Three-layer Graphene-SiO_2_-Gold	60
[28]	0.49–1.47 THz	100%	Monolayer graphene-SiO_2_-Au	-
Our work	0.80–1.87 THz	80.15%	Monolayer Graphene-polyimide-Au	60

## Data Availability

All data that support the findings of this study are included within the article.
